# PyCoTools: a Python toolbox for COPASI

**DOI:** 10.1093/bioinformatics/bty409

**Published:** 2018-05-22

**Authors:** Ciaran M Welsh, Nicola Fullard, Carole J Proctor, Alvaro Martinez-Guimera, Robert J Isfort, Charles C Bascom, Ryan Tasseff, Stefan A Przyborski, Daryl P Shanley

**Affiliations:** 1 Institute for Cell and Molecular Biosciences, Newcastle University, Newcastle, UK; 2 Institute of Cellular Medicine, Newcastle University, Newcastle, UK; 3 Department of Biosciences, Durham University, Durham, UK; 4 The Proctor & Gamble Company, Cincinnati, USA

## Abstract

**Motivation:**

COPASI is an open source software package for constructing, simulating and analyzing dynamic models of biochemical networks. COPASI is primarily intended to be used with a graphical user interface but often it is desirable to be able to access COPASI features programmatically, with a high level interface.

**Results:**

PyCoTools is a Python package aimed at providing a high level interface to COPASI tasks with an emphasis on model calibration. PyCoTools enables the construction of COPASI models and the execution of a subset of COPASI tasks including time courses, parameter scans and parameter estimations. Additional ‘composite’ tasks which use COPASI tasks as building blocks are available for increasing parameter estimation throughput, performing identifiability analysis and performing model selection. PyCoTools supports exploratory data analysis on parameter estimation data to assist with troubleshooting model calibrations. We demonstrate PyCoTools by posing a model selection problem designed to show case PyCoTools within a realistic scenario. The aim of the model selection problem is to test the feasibility of three alternative hypotheses in explaining experimental data derived from neonatal dermal fibroblasts in response to TGF-β over time. PyCoTools is used to critically analyze the parameter estimations and propose strategies for model improvement.

**Availability and implementation:**

PyCoTools can be downloaded from the Python Package Index (PyPI) using the command ’pip install pycotools’ or directly from GitHub (https://github.com/CiaranWelsh/pycotools). Documentation at http://pycotools.readthedocs.io.

**Supplementary information:**

Supplementary data are available at *Bioinformatics* online.

## 1 Introduction

In biology, systems modelling is used to reproduce the dynamics of a biochemical network of molecular interactions with a mathematical model. It has proved particularly useful in the study of cell signalling systems such as NF-κB (Adamson *et al.*, 2016; Ashall *et al.*, 2009; Nelson *et al.*, 2004), mTOR (Dalle Pezze *et al.*, 2012, 2016), p53 (Purvis *et al.*, 2012; Sun *et al.*, 2011) and TGF-β (Schmierer *et al.*, 2008; Vilar *et al.*, 2006; Wang *et al.*, 2014; Zi and Klipp, 2007; Zi *et al.*, 2014). In these studies, the essential biological relationships are represented by a series of ordinary differential equations (ODE) to generate a model. Hypotheses can then be tested by performing *in-silico* experiments. Before ODE models can be used to make meaningful predictions they must first be calibrated to experimental data.

Model calibration is a notoriously difficult problem typically due to the size and complexity of the systems involved and a lack of appropriate experimental data. ODE models are prevalent in systems biology because they are well-suited for predicting system dynamics and because many computational tools have been developed explicitly for the construction, simulation and analysis of biological networks. Among these tools are Data2Dynamics (Raue *et al.*, 2015), Systems Biology Workbench (Sauro *et al.*, 2003), AMIGO (Balsa-Canto and Banga, 2011), SBpipe (Dalle Pezze and Le Novère, 2017), libRoadRunner (Sauro *et al.*, 2013; Somogyi *et al.*, 2015), Antimony (Smith *et al.*, 2009), Tellurium (Choi *et al.*, 2016), Ecell (Takahashi *et al.*, 2003), PyDsTool (http://www2.gsu.edu/∼matrhc/PyDSTool.htm), PySCeS (Olivier, 2005), ABC-SysBio (Liepe *et al.*, 2010), Condor Copasi (Kent *et al.*, 2012) and COPASI (Hoops *et al.*, 2006).

COPASI is a widely used tool in modelling biological systems because it supports a variety of modelling applications including deterministic, stochastic and hybrid model solvers, parameter estimation, optimization, parameter scans, steady state analysis, local sensitivity analysis and metabolic control analysis. COPASI has a graphical user interface (GUI) which makes the tool accessible to non-expert programmers and mathematicians, but also has a command line interface for batch processing and an application programming interface (API) for several programming languages. These APIs have been used for integrating the COPASI framework with custom software, for example in JigCell Run Manager (Palmisano *et al.*, 2015), CellDesigner (Matsuoka *et al.*, 2014), ManyCell (Dada and Mendes, 2012) and ModelMage (Flöttmann *et al.*, 2008).

The Python programming language is useful for scientific computing because of its concise syntax and the availability of open source toolboxes such as pandas (https://pandas.pydata.org/), numpy (http://www.numpy.org/), scipy (http://www.scipy.org/), sklearn (Pedregosa *et al.*, 2011) and matplotlib (Hunter, 2007), which together provide a series of well-documented, easy-to-use, high-level tools for interacting with and manipulating numerical data. Development of further tools in Python is enabled by the Python Package Index (PyPI) where code can be made freely available to other developers. As a result, Python has an extensive publicly available code base for scientific computing that competes well with other commercial and non-commercial environments such as Matlab and R.

Here we present PyCoTools, an open-source Python package which provides a high level interface to COPASI tasks with an emphasis on model calibration. COPASI tasks are integrated with the Python environment to provide additional features which are non-native to COPASI. Features include: the construction of COPASI models with Antimony (Smith *et al.*, 2009); the automation of repeat parameter estimation configurations, chaser parameter estimations and parameter estimations for multiple models (e.g. model selection); automation of the profile likelihood method of identifiability analysis (Raue *et al.*, 2013; Schaber, 2012) with visualization facilities which are flexible enough to support model reduction (Maiwald *et al.*, 2016); visualization of time courses from ensembles of parameter sets and multiple ways of visualizing parameter estimation data. We demonstrate PyCoTools by defining a model selection problem to introduce a known negative feedback into a previously published model of TGF-β signalling (Zi and Klipp, 2007) using new data.

## 2 Materials and methods

### 2.1 Experimental

#### 2.1.1 Cell lines and treatment

Neonatal human dermal fibroblasts (HDFn, Life Technologies, C-004-5C) were cultured as per manufacturer guidelines in M106 (Life Technologies M-106-500) supplemented with LSGS (Life Technologies S-003-10). HDFn were seeded at a density of 10 000 cells/cm^2^ into 12 well plates (Greiner 665180) in 4 ml complete M106 and cultured for 3 days. Media was aspirated, cells washed twice with DPBS and replaced with 4 ml M106 without LSGS and cells were serum starved for 24 h. HDFn were treated with 5 ng ml^–^^1^ TGF-β1 (Life Technologies, PHG9211) in M106 media without LSGS for 0, 1, 2, 4, 8, 12 h. To harvest, media was aspirated, cells were washed twice in DPBS and then lysed in 350 µl RLT buffer (Qiagen 79216).

#### 2.1.2 High-throughput qPCR

Lysates were snap frozen in liquid nitrogen and stored -80°C prior to quantification. Cell lystes were thawed at 4°C and then RNA was isolated using the Biomek FxP and the RNAdvance Tissue Isolation kit (Beckman Coulter, p/n A32646). The resulting RNA was quantified using the Nandrop 8000 (Nanodrop, ND-8000). cDNA was generated using 500 ng of TotalRNA and Applied Biosystems High Capacity cDNA with Reverse Transcription kit (Applied Biosystems p/n 4368814). cDNA, assays and dilutions of Applied Biosystems Taqman Fast Advanced MasterMix (Applied Biosystems, p/n 4444965) were plated onto a Wafergen MyDesign SmartChip (TakaraBio, p/n 640036) using the Wafergen Nanodispenser. The chip was then loaded into the SmartChip cycler and qPCR performed using the following conditions: hold Stage 50°C for 2 min, 95°C for 10 min, PCR Stage 95°C for 15 s and 60°C for 1 min. After 40 cycles the reaction was stopped and the data was exported for analysis.

Prior to use for fitting, cycle threshold *C_T_* values were normalized using the 2^−ΔΔC_T_^ method of quantitative PCR normalization to the geometric mean of four reference genes (B2M, PPIA, GAPDH, ACTB) per sample (Livak and Schmittgen, 2001).

### 2.2 Computational

#### 2.2.1 PyCoTools availability and installation

PyCoTools was developed partially on Windows 7 and partially on Ubuntu 16.04.2 with the Anaconda distribution of Python 2.7 and COPASI version’s 4.19.158 and 4.21.166. PyCoTools can be installed with ‘pip’, Python’s native package manager using the command ‘pip install pycotools’. PyCoTools can also be downloaded directly from source at https://github.com/CiaranWelsh/pycotools. More detailed instructions on installation and PyCoTools usage can be found in the PyCoTools documentation (http://pycotools.readthedocs.io).

#### 2.2.2 Definition of the model selection problem

All models were built by downloading the Zi and Klipp (2007) model from BioModels (ID: BIOMD0000000163) and modifying it as appropriate using the COPASI user interface for each model. The models are available in the supplementary content as SBML files. Model selection was performed by calibrating each model to the same experimental data and then evaluating model selection criteria. The Ski mRNA and Smad7 mRNA profiles were measured whilst protein level data were derived by assuming that Smad7 and Ski protein appear 30 min after the mRNA and at 100 times the magnitude. Since the experimental data units are arbitrary and the Zi and Klipp (2007) model simulates in nanomoles per litre, the experimental data were mapped to the model via an observation function (Equation 1).

(1) $$X_{Obs_{\left(t\right)}}=\frac{X_{\left(t\right)}}{X_{SF}}$$

where:

$$\begin{array}{l} X_{Obs_{\left(t\right)}}=\text{A mapping between experimental and simulated data}\\ X_{\left(t\right)}=\text{Amount of model species X at time t}\\ X_{SF}=\text{Scale factor for species X}=100\\ X\in \{\text{Smad7mRNA},\text{SkimRNA},\text{Smad7Protein},\text{SkiProtein}\} \end{array}$$

All scale factors were set to 100 which is a reasonable value to ensure new profiles were of the same order of magnitude as the original. The initial concentration of Smad7 and Ski protein were set to 100 times that of the corresponding mRNA and all new kinetic parameters were estimated. All parameters from the original Zi *et al.* (2014) model were fixed at the published values, including initial concentration parameters. Initial concentrations of Smad7 mRNA and Ski mRNA were set using Equation 2:

(2) $$X_{\left(t0\right)}=X_{\left(\mu ,t0\right)}\cdot X_{SF}$$

where:

$$\begin{array}{c} X_{\left(t0\right)}=\text{Initial}\;\text{amount}\;\text{of}\;\text{species}\;X\;\text{in}\;\text{the}\;\text{model}\\ X_{\left(\mu ,t0\right)}=\text{Empirical}\;\text{average}\;\text{of}\;\text{species}\;X\;\text{at}\;t=0\;\text{in}\;\text{arbitrary}\;\text{units} \end{array}$$

All parameters were estimated between the boundaries of $1e^{-7}$ and $1e^{4}$. Three hundred parameter estimations were performed per model using COPASI’s stochastic genetic algorithm with a population size of 300 over 500 generations and starting from random values. The residual sum of squares (RSS) objective function was weighted using the standard deviation of the 6 data replicates. All parameter estimations were configured and run simultaneously using PyCoTools ‘tasks.MultiModelFit’ class on a computer cluster running the Sun-Grid Engine job scheduling software. The estimations can optionally be configured to run on a single machine.

#### 2.2.3 An idealized model selection problem

In addition to the main model selection demonstration, another idealized model selection demonstration has been provided in the supplementary content. The purpose of this alternative demonstration is to provide an example with short execution times that parallels the main model selection problem and provides code that users can run themselves. Specifically, in this alternative model selection problem we create three models (a negative feedback motif, a positive feedback motif and a feed-forward motif) using the Antimony interface. Analogous to the main problem defined above, we then perform model selection using synthetic experimental data from the negative feedback topology, visualize the results and run an identifiability analysis.

## 3 Results

### 3.1 Overview of PyCoTools facilities and architecture

PyCoTools provides COPASI users with a means of efficiently configuring and running COPASI tasks from a Python environment. The PyCoTools package is comprised of three main modules: ‘model’, ‘tasks’ and ‘viz’.

The ‘Model’ object under the ‘model’ module plays a central role in PyCoTools by using Python’s ‘lxml’ library to extract model information from the COPASI XML and store it in Python classes. Manipulating XML was chosen because of its widespread use in systems biology and because well documented tools exist for its manipulation. The information extracted is subsequently available as ‘Model’ attributes. The ‘Model’ enables users to add, remove and change model components and acts as a central entity that can be modified and configured by other PyCoTools classes. As an alternative means of building models, the ‘model’ module provides an interface to and from the SBML model definition language, Antimony (Smith *et al.*, 2009). PyCoTools wraps functions from Tellurium (Choi *et al.*, 2016) and command line COPASI to convert between Antimony, SBML and COPASI models, thereby facilitating the transition between environments.

The ‘tasks’ module uses the ‘Model’ class extensively to configure COPASI tasks. Supported tasks include deterministic, stochastic or hybrid time courses, arbitrary dimensional parameter scans or repeat tasks, and parameter estimations. Additionally, tasks are provided which are not available in COPASI within a single function. Specifically, PyCoTools automates the configuration of ‘repeat parameter estimations’ and increases the rate by which parameter estimations can be run. This is achieved by automatically configuring COPASI’s repeat parameter estimation feature and running model replicates simultaneously. A queueing system is introduced to prevent overuse of limited computational resources. PyCoTools supports the configuration and running of ‘chaser estimations’ where parameter estimates from a global algorithm are inserted into the model and driven to a minimum with a local algorithm. Other tasks supported by PyCoTools include model selection and the calculation of profile likelihoods for assessing a identifiability status of a model (Raue *et al.*, 2009; Schaber, 2012).

The ‘viz’ module [the concept of which takes inspiration from the Ecell software by Takahashi *et al.* (2003)] contains all PyCoTools visualization facilities. The aim of the ‘viz’ module is to produce publication quality figures of time courses, parameter estimations, profile likelihoods and model selection. The ‘viz’ module also provides a host of exploratory data analysis tools for analyzing repeat parameter estimation data. These tools and their usage are described next.

#### 3.1.1 Tools for analysis of repeat parameter estimation data

Repeat parameter estimation data can be visualized in multiple ways and this information can be used to diagnose problems and direct modelling efforts. The tools provided in PyCoTools collectively allow one to gauge uncertainty in model predictions or parameter estimates, assess the performance of algorithms used for optimization, visualize distributions of parameters and visualize putative relationships between parameters.

Usually the first item of interest after a parameter estimation is to visualize simulated predictions against empirical data. PyCoTools extends the basic ‘simulated versus experimental time course plot’ to calculate and display confidence intervals for each profile. This is achieved by inserting parameter sets into the model in turn, simulating a time course and aggregating the results by bootstrapping an estimator (e.g. the mean) of the users choice. By visualizing predictions from several parameter sets, uncertainty is propagated from parameter estimates to model predictions. The ‘ensemble time course’ thus emphasizes model strengths and weaknesses, highlighting regions of confidence and those which require attention.

While ensemble time courses are used to inform our confidence on model predictions, profile likelihoods are used to inform our confidence on parameter values. Briefly, a profile likelihood is a parameter scan of parameter estimations, starting from a best parameter set. Each parameter is fixed in turn and its value is systematically varied over the course of the scan. The remaining parameters are re-optimized at each point of the scan and the objective function value traces a path through parameter space. The shape of this profile is then compared to a confidence threshold based on the likelihood ratio statistic (Raue *et al.*, 2009).

A profile likelihood typically has one of three interpretations. If the profile does not exceed the threshold in one or both directions and is not flat, the parameter is practically non-identifiable. In this case, the trajectory of the other model components over the profile may be used to direct model reduction strategies (Maiwald *et al.*, 2016). If a profile is completely flat the parameter is structurally non-identifiable, which means the parameter is algebraically related to another. To resolve structural non-identifiabilities, one can fix one of the parameters in a relationship to an arbitrary value. Of note, one must be cautious about using profile likelihoods to render a parameter structurally non-identifiable because the profile likelihood method only samples the parameter space. It is possible that the profile appears flat but only on the scale of the sampled profile. Therefore, structurally non-identifiable parameters should be further investigated to determine any relationships which might exist. Finally, if the profile exceeds this threshold in both directions the parameter is identifiable and the parameter values at which the profile exceeds the threshold are the upper and lower confidence boundaries for the parameter (Raue *et al.*, 2009). Ideally, for precise model predictions, every estimated parameter in a defined parameter estimation problem should be identifiable. In reality, limited data and overly complex model structures often lead to identifiability issues.

Maiwald *et al.* (2016) extended the usefulness of profile likelihood from assessing identifiability to model reduction. A practical non-identifiability exists because the optimization does not have enough data to inform model parameters, or put another way, the model is too complex for the data. Viewing the paths traced by other parameters in a profile likelihood analysis (e.g. putting the trajectory of another parameter on the y-axis rather than the objective function value) provides information about the relationship between the parameter of interest on the x-axis and the parameter on the y-axis. Identifying this relationship enables steps to be taken to resolve the problem by fixing parameters or replacing non-identifiable species or parameters with algebraic equations. Profile likelihoods are therefore useful in a data-driven approach to iteratively refine an optimization problem, fixing parameters where possible and modifying the topology as necessary until the model fits the experimental data.

Profile likelihood calculations are a computationally intense task and to be useful, it is required that the starting parameter set is optimal, or at least very close to optimal, with respect to the data. It is therefore prudent to assess this condition before conducting a profile likelihood analysis. The performance of an optimization problem can be evaluated by plotting the sorted objective function value [i.e. residual sum of squares (RSS) or likelihood] for each parameter estimation iteration against its rank of best fit (herein referred to as a ‘likelihood-ranks’ plot). In these plots the best case scenario is either a flat line for when there is only a single global minimum or more commonly, a monotonically increasing step-like function where each step marks a different minimum (Raue *et al.*, 2013). Horizontal lines in the likelihood-ranks plot indicate that many iterations of the same optimization problem have located the same minimum, which increases our confidence that the problem is well-posed. In contrast a smooth curve indicates that estimations have not converged to a minimum.

If the likelihood-ranks plot shows a smooth curve, it is a good idea to either rerun the parameter estimation using a different algorithm or different algorithm settings. Alternatively, while others (Raue *et al.*, 2013) employ a multi-start Latin-hypercube strategy with a local optimizer to ensure strategic and uniform sampling of the parameter space, given the choice of algorithms in COPASI it is easy to first run a global and then switch to a local algorithm. This strategy, here referred to as a ‘chaser estimation’, can be performed on all or a subset parameter sets to drive them closer to their respective minima.

In addition to profile likelihoods and time course ensembles, viewing distributions of parameter estimation data and correlations between parameters can provide information about an optimization problem. Box plots provide immediate information about the range of parameter estimates and how they compare to other parameters. Often a box plot can provide clues to a parameter’s identifiability status. Histograms on the other hand provide a more detailed view of parameter distributions and can identify behaviour (e.g. bimodal parameters) that would not be identified with box plots. Moreover, a combination of Pearson’s correlation heat maps and scatter graphs can be used to locate linear or log-linear relationships between parameters.

An important aspect of visualizing parameter estimation data is that not all parameter sets fit the model equally well. Parameter sets with higher objective function values can distort the distribution of better performing parameter sets or the shape of a relationship. For this reason PyCoTools implements flexible means of subsetting parameter estimation data before plotting.

### 3.2 A demonstration: extending the Zi and Klipp (2007) model

**Fig. 1.**
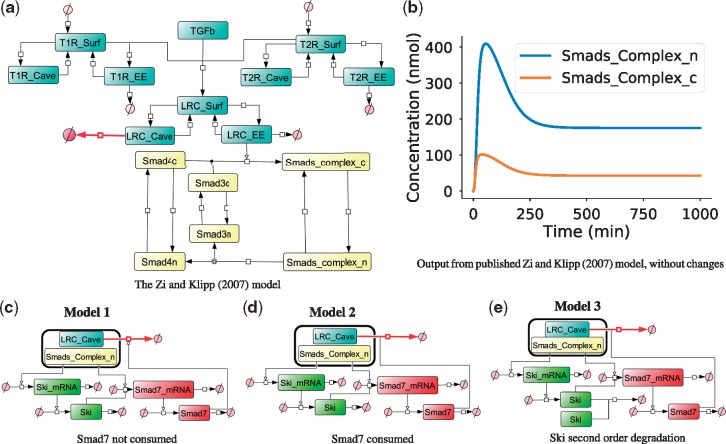
Network representation of ODE networks used in model selection problem. (**a**) The Zi and Klipp (2007) model is a common component of each model variant. (**b**) Simulation output from the Zi and Klipp (2007). (**c–e**) The model variable ‘Smads_Complex_n’ is responsible for transcription reactions in model variants while ‘LRC_Cave’ is degraded by Smad7 protein, thus completing the explicit representation of the Smad7 negative feedback loop. In (c) Model 1, Smad7 participates in but is not consumed by the reaction with LRC_Cave while in (d) Model 2, Smad7 is consumed by this process. In (e) Model 3, the same topology as Model 2 is assumed but it also incorporates second order mass action degradation kinetics for Ski protein

To demonstrate PyCoTools, we define a model selection problem to extend a published model of canonical TGF-β signalling (Zi and Klipp, 2007) (Fig. 1). As an alternative demonstration, we also provide an another model selection problem in the supplementary content, as described in the methods.

TGF-β binds to the autophosphorylated homodimeric type 2 TGF-β receptors which phosphorylate and heterodimerize with homodimers of type 1 TGF-β receptors (De Crescenzo *et al.*, 2001). This event leads to internalization of the ligand–receptor complex into one of two types of membrane bound intracellular compartment: early endosomes or caveolae. Evidence in Di Guglielmo *et al.* (2003) suggests that ligand–receptor complexes in the early endosome, rather than the caveolae, are responsible for conveying the TGF-β signal, via phosphorylation, to the Smad second messenger system. Phosphorylated Smad2/3 binds to Smad4, translocates to the nucleus and induces transcription of TGF-β responsive genes (Schmierer *et al.*, 2008). Smad7 is a well characterized negative regulator of the Smad system and is transiently produced in response to TGF-β (Hayashi *et al.*, 1997; Nakao *et al.*, 1997). Multiple mechanisms of negative regulation by Smad7 have been reported, including the recruitment of E3 ubiquitin ligases to either Smad2/3 in competition with Smad4 (Yan *et al.*, 2016) or to activated TGF-β receptors in caveolae (Di Guglielmo *et al.*, 2003; Kavsak *et al.*, 2000). Many biological entities have been proposed as regulators of this process, including PPM1A (Lin *et al.*, 2006), NEDD4L (Gao *et al.*, 2009), SNoN (Stroschein *et al.*, 1999) and Ski. Ski acts as co-repressor at Smad regulated genes by recruiting histone deacetylases which leads to epigenetic constriction of Smad-responsive genes (Akiyoshi *et al.*, 1999).

**Fig. 2.**
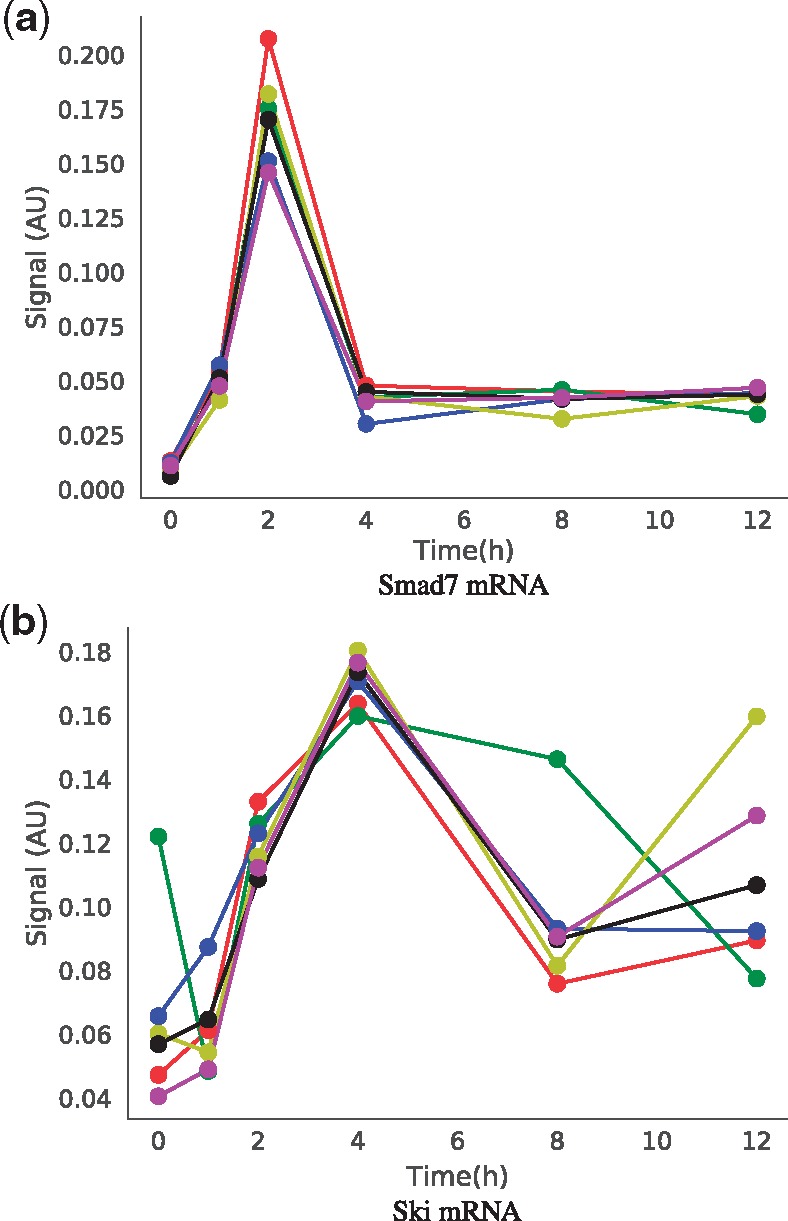
Experimental data used for model calibration. Neonatal human dermal fibroblasts were treated with 5 ng ml^–1^ TGF-β for 0, 1, 2, 4, 8 and 12 h. Shown are profiles of 6 biological replicates for (**a**) Smad7 and (**b**) Ski messenger RNA, measured by high throughput quantitative PCR as described in the methods

The Zi and Klipp (2007) model (Fig. 1a) combines work by Vilar *et al.* (2006) describing TGF-β receptor internalization and recycling dynamics with a Smad nuclear-cytoplasmic translocation module. In this model, an explicit representation of the Smad7 negative feedback was not included, but was instead incorporated into the rate law for the reaction describing the degradation of the activated ligand–receptor complexes from within caveolar compartments (‘LRC_Cave’ in Fig. 1a). The purpose of the model selection problem presented here is to investigate the feasibility of three alternative mechanisms of negative regulation (Fig. 1) in explaining the experimental data (Fig. 2).

**Fig. 3.**
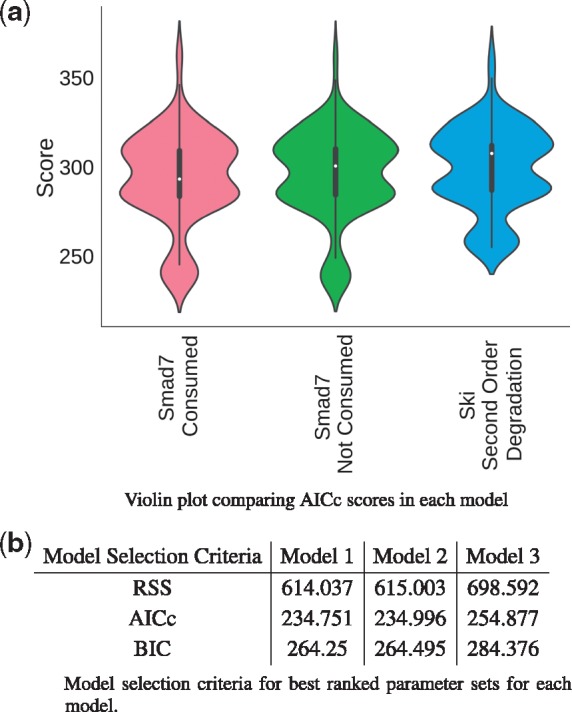
Model selection criteria. (**a**) Distribution of Akaike information criteria (AICc) per model displayed as violin plot. The central white dot represents the median; the thin centre line is the 95% confidence interval; the thick central bar is the interquartile range and the width represents the frequency with which a score was observed. These graphs were produced with ‘viz.ModelSelection’. (**b**) A comparison of model selection criteria for the best ranking parameter sets in each model

After calibration, the ‘viz.ModelSelection’ class was used to calculate and visualize the Akaike information criteria (AIC) corrected for small sample sizes (AICc) (Fig. 3a) and the Bayesian information criteria (BIC) (Supplementary Fig. S1). With these statistics, a lower value indicates a better agreement with the data and thus a better model. In the current problem, a closer inspection of the best model selection values (Fig. 3b) indicates that from a purely statistical perspective, the topologies of Models 1 and 2 are indistinguishable in terms of the experimental data (Fig. 2) while Model 3 is worse.

**Fig. 4.**
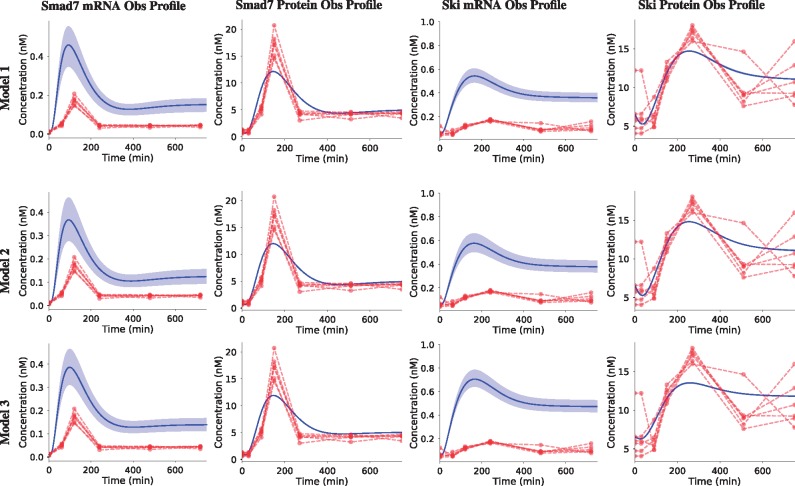
Ensemble time courses produced with ‘viz.PlotTimeCourseEnsemble’. The top 10 best parameter sets for each model were sequentially inserted into their respective models. Time courses were simulated with each parameter set and averaged. Red profiles indicate experimental data while solid blue lines are simulated profiles. Shaded areas represent 95% confidence intervals

The simulated profiles for each model (Fig. 4) supports the model selection results. While the Smad7 mRNA and Ski mRNA profiles are slightly greater in Model 1 and Model 3 respectively, all profiles are virtually indistinguishable between all the models. It is likely that the difference in the Ski mRNA profile in Model 3 accounts for the difference observed in the best model selection criteria (Fig. 3b). Regardless of this slight difference, the same qualitative interpretation holds for each model: the speed and magnitude of both Smad7 and Ski mRNA induction profiles are overestimated while the protein level data fits each model to a high degree of confidence.

**Fig. 5.**
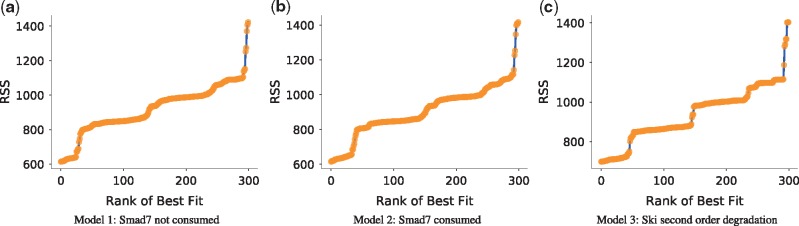
A ‘likelihood-ranks’ plot. The residual sum of squares objective function value is plotted against the rank of best fit for each parameter estimation iteration for each model (**a–c**). Graphs were produced with ‘viz.LikelihoodRanks’

When looking at model predictions it is important to consider whether the parameter sets used to produce them are actually the best parameter sets. This is important because it is quite common for parameter estimation algorithms to find sub-optimal parameters. Here, while improvements can still be made, the algorithm and settings were reasonably well-chosen because the likelihood-ranks plot produced a step-like shape for each model (Fig. 5), heuristically mapping out where the local and global minima are.

**Fig. 6.**
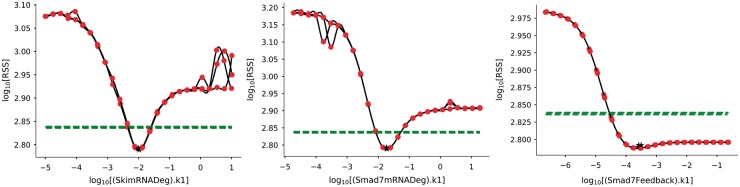
Profile likelihoods were calculated using the ‘tasks.ProfileLikelihood’ class for the top three parameter sets of Model 2 and visualized using ‘viz.PlotProfileLikelihood’. The black stars indicate the best estimated parameter. The dotted green line indicates the 95% confidence level and the red spots are the minimum RSS value achieved after re-optimization of all parameters except the parameter of interest (x-axis). Lines between red spots have been interpolated using a cubic spline

Profile likelihoods are only meaningful when calculated from a minimum with respect to the data. For this reason the best three parameter sets from the stochastic genetic algorithm in Model 2 were ‘chased’ with a Hooke & Jeeves algorithm (tolerance = 1$e^{-10}$ and iteration limit = 1000) using the ‘PyCoTools.tasks.ChaserParameterEstimations’ class. Profile likelihoods were then computed around these three parameter sets, again using the Hooke & Jeeves algorithm (tolerance = 1$e^{-6}$ and iteration limit = 50). Sampling was conducted on a log10 scale over 6 orders of magnitude, 1e^3^ times above and below the best estimated parameter values. For brevity, profile likelihoods for Models 1 and 3 are not discussed. The identifiability analysis shows that seven of the ten parameters are identifiable and the remaining three are practically non-identifiable (Fig. 6 and Supplementary Fig. S2).

**Fig. 7.**
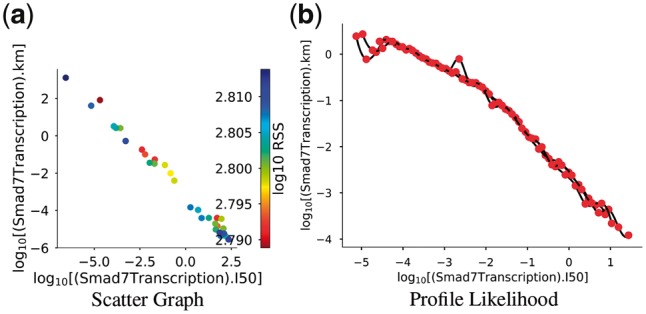
Identification of a log-linear relationship between ‘(Smad7Transcription).km’ and ‘(Smad7Transcription).I50’ (*k_m_* and *I*_50_, respectively). (**a**) Scatter graph showing that as *k_m_* increases, *I*_50_ decreases (r^2^ = 0.995, *P*-value = 1$e^{-39}$). (**b**) The path traced by *k_m_* is plotted as a function of *I*_50_ during the profile likelihood calculation. Graphs were produced using ‘viz.Scatters’ and ‘viz.PlotProfileLikelihood’ respectively

To investigate the source of these non-identifiabilities, two strategies were employed: Pearson’s correlation analysis and the ‘profile likelihood model reduction’ approach as described in Maiwald *et al.* (2016). The Pearson’s correlation approach identified several parameter pairs as putative linear correlations (Supplementary Fig. S3). Of these, only the most correlated pair, the *k_m_* and *I*_50_ parameters of Smad7 transcription, was verified to be log-linearly related in both scatter graphs (Fig. 7a) and profile likelihood traces (Fig. 7b). To resolve this issue, one could replace one of the free parameters in the relationship with the algebraic equation resulting from the fit of a linear model to the profile likelihood trace (Fig. 7b). The other putative relationships suggested by the Pearson’s correlation analysis (Supplementary Fig. S3) were also investigated but the relationships were more difficult to interpret. As an example, Supplementary Figure S4 shows the relationship between ‘(SkiDeg).k1’ and ‘(SkimRNADeg).k1’ parameters. While the scatter graph shows a reasonable linear correlation (Supplementary Fig. S4a), it is defined on a very small interval and the profile likelihood is clearly non-linear, albeit linear on a sub-domain of the parameter space (Supplementary Fig. S4b).

Lastly, distributions of parameter estimates were visualized using box plots (Supplementary Fig. S5) and histograms (Supplementary Fig. S6). Despite being presented last, these are computationally inexpensive to generate and are good to view prior to more involved analyses such as profile likelihoods. To demonstrate the effect of sub-optimal parameter sets, a comparison is made between box plots generated for Model 2 using all parameter estimation data (Supplementary Fig. S5a) to those using only the top 10% ranking parameter sets (Supplementary Fig. S5b). Supplementary Figure S5 demonstrates that suboptimal parameter sets can distort the insight that can be gained from visually exploring parameter estimation data. Without truncating the parameter estimation data, the observation that the distributions of parameters from the best parameter sets reflect the identifiability status of the model, would be missed.

## 4 Discussion

PyCoTools is an open source Python package designed to assist COPASI users in the task of modelling biological systems. PyCoTools offers an alternative high level interface to COPASI tasks including time courses, parameter scans and parameter estimations. While COPASI implements the heavy computation, PyCoTools automates task configuration and execution, thereby promoting efficiency, organization and reproducibility.

PyCoTools bridges COPASI with the Python environment allowing users to take advantage of Python’s numerical computation, visualization, file management and code development facilities. One tool in particular, the Jupyter notebook, allows annotation of code blocks with rich text elements and is a powerful environment from which to develop and share annotated workflows. The combination of Jupyter notebooks, COPASI and PyCoTools therefore enables the production of reproducible and shareable models that are annotated with justifications.

PyCoTools supports model editing using both an object-oriented approach and with Antimony, a model specification language for building SBML models (Smith *et al.*, 2009). The Antimony and COPASI user interface are complementary and can be used together to enhance the modelling process. For example, models in Antimony format can be used as a ‘hard copy’ while a parallel COPASI model can be used for exploratory changes that are ‘committed’ to the hard copy when satisfactory.

PyCoTools supports the configuration of ‘composite’ tasks which are those comprised of a combination of other tasks. These tasks can be configured using the COPASI user interface but generally take time and are vulnerable to human error. For example, users can automatically configure repeat parameter estimations, chaser parameter estimations and model selection problems, thereby circumventing the requirement for manual configuration.

Another composite task supported by PyCoTools is the profile likelihood method of identifiability analysis (Raue *et al.*, 2009). Models with non-identifiable parameters are common in systems biology and it is useful to have a means of assessing which parameters are reliably defined by an estimation problem. PyCoTools automates the procedure outlined by Schaber (2012) for conducting profile likelihoods in COPASI, thereby enabling COPASI users to perform an identifiability analysis more efficiently and in a way less amenable to errors than manual configuration. PyCoTools also enables users to calculate profile likelihoods from multiple parameter sets thereby enabling users to address one of the shortcomings of the profile likelihood approach: that it is a local method of identifiability analysis.

One alternative to COPASI and PyCoTools is Data2Dynamics (Raue *et al.*, 2015). While Data2Dynamics provides an excellent range of model analysis tools, the transfer of files between COPASI and Data2Dynamics is imperfect, often necessitating that a COPASI user redefine their model within the Data2Dynamics environment. PyCoTools allows COPASI users to stay within the COPASI environment, thereby making profile likelihood analysis more accessible to COPASI users.

In this work we have demonstrated PyCoTools by posing a model selection problem to discriminate between three model topologies (Fig. 1) with respect to some experimental data in response to TGF-β (Fig. 2). Rather than using synthetic data, our aim was to demonstrate in a ‘real world’ scenario how PyCoTools can be used together with COPASI to calibrate a set of models and discriminate between them.

As this is primarily a software demonstration and not a biological investigation, the model selection problem proposed was designed to be as simple as possible whilst still being non-trivial. Mechanistically the three models (Fig. 1) are alternative hypotheses which attempt to address the dynamics of the Smad7 (Fig. 2) negative feedback. Model alternatives were based on a published dynamic model of TGF-β signalling (Zi and Klipp, 2007) that was adapted to incorporate Smad7. Since the decay of Smad7 is transient and fast (Fig. 2a), the simplest mechanism involving only Smad7 with first order mass action degradation kinetics would not be able to account for the observed decline in Smad7. Therefore Smad7 degradation was assumed to be an active process. Since Ski is a known Smad co-repressor (Akiyoshi *et al.*, 1999) and Smad7 is a Smad responsive gene (Hayashi *et al.*, 1997), Ski was proposed to be transcribed in response to TGF-β (Fig. 2b) and inhibit Smad7 transcription. The model alternatives are slightly different representations of this hypothesis (Fig. 1).

In this model selection problem it is clear that the model topologies chosen are too similar to be discriminated with the experimental data and therefore the models are virtually indistinguishable (Fig. 4). Generally, with model selection, the strongest statement that can be made about a model is a rejection, since accepting the hypothesis does not necessarily guarantee that it is correct. By comparing the performance of multiple models in model calibration it is possible reject one or more topologies in favour of another. Here, however, because the models are so similar, it was not possible to provide support for any model being worse than any other, despite the minor differences in model selection criteria for Model 3 (Fig. 3a). In a more comprehensive investigation many more topologies would be similarly compared to iteratively reject topologies until the model is capable of making useful, validatable predictions.

Regardless of the biological interpretation, we have demonstrated the process of using PyCoTools and COPASI to discriminate between model alternatives and to critically assess the parameter estimation process. Model calibration is an essential part of a systems modelling investigations, but it is often limited by a vast, underdetermined parameter space and therefore, procedures that provide a measure of uncertainty are valuable. In PyCoTools, we have implemented a number of features aimed towards gauging confidence and uncertainty in the optimization process so that COPASI users can diagnose problems and make better informed decisions based on their parameter estimation output. These tools include: the likelihood-ranks plot (Fig. 5) which enables evaluation of an optimization algorithm and settings on a specific problem (Raue *et al.*, 2013); ensemble time courses (Fig. 4) which calculate confidence intervals from predictions made from multiple best parameter sets and propagates uncertainty from parameter estimates to model predictions; profile likelihoods for assessing identifiability (Fig. 6, Supplementary Fig. S2) and for model reduction (Fig. 7b) (Maiwald *et al.*, 2016); Pearson’s correlation heat maps (Supplementary Fig. S3) and scatter graphs (Fig. 7a) for identifying relationships, and box plots (Supplementary Fig. S5) and histograms (Supplementary Fig. S6) for visualizing distributions of parameter estimates. Together these tools provide detailed information about an optimization problem that can be used to guide the modelling process.

## 5 Conclusion

PyCoTools is an open-source and extensible Python package designed to facilitate the use of COPASI, particularly for model calibration. PyCoTools supports a range of tools which are either wrappers around COPASI tasks, an ordered workflow of task configurations, or plotting facilities for exploratory data analysis on parameter estimation data. Use of PyCoTools can enhance the effectiveness with which one can calibrate models to experimental data and discriminate between alternate hypotheses.

## Funding

This work was funded by Procter & Gamble. The contribution from AGM and CJP was supported by the Medical Research Council (https://www.mrc.ac.uk/) and Arthritis Research UK (http://www.arthritisresearchuk.org/) as part of the MRC-Arthritis Research UK Centre for Integrated research into Musculoskeletal Ageing (CIMA) (MR/K006312/1). The work builds on a BBSRC LINK grant awarded to SAB (BB/K019260/1).

*Conflict of Interest*: none declared.

## Supplementary Material

Supplementary Data
