# Supplementary material for: PyCoTools: a Python toolbox for COPASI
Source: Bioinformatics. 2018 May 22;34(21):3702–10. doi: 10.1093/bioinformatics/bty409 (PMC6198863; doi:10.1093/bioinformatics/bty409)
Supplement: Supplementary Data [file bty409_supp.zip › bty409-suppl_data/WelshBioinformatics revised Supplementary with Tutorial.pdf]

Welsh et. al., PyCoTools: A Python Toolbox for COPASI. Supplementary Content: A PyCoTools Session

April 23, 2018

1 PyCoTools Example Session

In the main text, we illustrate the use of PyCoTools by working through a model selection problem using experimental time series data to discriminate between three alternative model topologies. The outcome was that the data was insufficient to discriminate between them and that the models were too similar.

Here, we provide an alternative example of an idealised model selection problem that was designed to be analogous to the problem in the main text but with more detail. These models are ideal for this demonstration because they are simple enough not to require a cluster of computers for simulation and the data is synthetic, e.g. simulated from one of the models. The latter ensures that there is a correct answer to the problem that can be used validate the results.

This document is designed to serve as a demonstration of a PyCoTools session but not to comprehensively describe all aspects of PyCoTools and users are referred to the documentation (http://pycotools.readthedocs.io) for more details.

In general, we provide tools for gaining insight into an optimization problem but we do not advocate a particular workflow that should be followed. All model calibrations are unique and some tools may be more useful in some situations than in others.

1.1 An Idealized Model Selection Problem

Shown below are three simple models with distinct topology. In this tutorial, synthetic data is simulated from the negative feedback model and used in a model selection problem to distinguish it from the others.

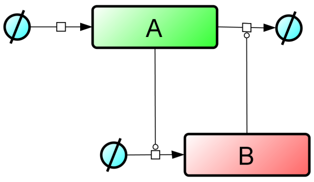

Negative Feedback Model

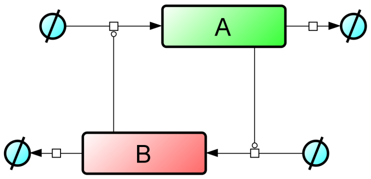

Positive Feedback Model

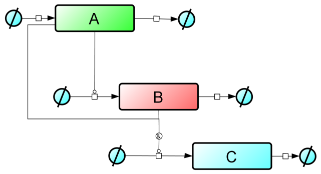

Feed Forward Model

1.2 A Broad Overview

To perform model selection using PyCoTools, all model and data files should be placed into a new directory. Data files should conform to the same format as the COPASI GUI (see http://copasi.

org/Support/User_Manual/Tasks/Parameter_Estimation/) with the additional requirement that column headers must match model variables exactly. The one exception is when independent variables are used in which case the suffix _indep should be used. It is advised that users do not use complicated naming conventions or non-ASCII characters.

The tasks.MultiModelFit class detects COPASI and data files (.txt or .csv) in the folder and sets up a multi-parameter estimation for each model using the tasks.MultiParameterEstimation class. After fitting, the viz.ModelSelection class is used to calculate and plot the AIC and BIC.

1.3 Model Definition with Antimony

For detailed instructions on how to define an antimony model, users are referred to the Tellurium documentation (http://tellurium.readthedocs.io/en/latest/antimony.html).

```
In [1]: import os, glob
        from pycotools import model, tasks, viz, misc
        import seaborn
        %matplotlib inline
        seaborn.set_context('notebook')

        working_directory = r'/home/b3053674/Documents/Models/2018/'\
                            '03_March/ModelSelectionDemonstration'

        ## create directory for our model selection analysis
        model_selection_dir = os.path.join(working_directory, 'ModelSelection')

        if not os.path.isdir(model_selection_dir):
            os.makedirs(model_selection_dir)

        ## create paths to three model alternatives.
        ## This should be inside a new clean directory
        copasi_file1 = os.path.join(model_selection_dir, 'negative_feedback.cps')
        copasi_file2 = os.path.join(model_selection_dir, 'positive_feedback.cps')
        copasi_file3 = os.path.join(model_selection_dir, 'feedforward.cps')

        ## build models with antimony
        with model.BuildAntimony(copasi_file1) as loader:
            mod1 = loader.load(
                """
                model negative_feedback
                    compartment cell = 1.0
                    var A in cell
                    var B in cell

                    vAProd = 0.1
                    kADeg = 0.2
                    kBProd = 0.3
                    kBDeg = 0.4
                    A = 0
```

```
            B = 0

            AProd: => A; cell*vAProd
            ADeg: A =>; cell*kADeg*A*B
            BProd: => B; cell*kBProd*A
            BDeg: B => ; cell*kBDeg*B
        end
        """
    )

with model.BuildAntimony(copasi_file2) as loader:
    mod2 = loader.load(
        """
        model positive_feedback
            compartment cell = 1.0
            var A in cell
            var B in cell

            vAProd = 0.1
            kADeg = 0.2
            kBProd = 0.3
            kBDeg = 0.4
            vBasalAProd = 0.001
            A = 0
            B = 0

            AProd: => A; cell*vAProd*B+vBasalAProd
            ADeg: A =>; cell*kADeg*A
            BProd: => B; cell*kBProd*A
            BDeg: B => ; cell*kBDeg*B
        end
        """
    )

with model.BuildAntimony(copasi_file3) as loader:
    mod3 = loader.load(
        """
        model feed_forward
            compartment cell = 1.0
            var A in cell
            var B in cell
            var C in cell

            vAProd = 0.1
            kADeg = 0.2
            kBProd = 0.3
            kBDeg = 0.4
            kCDeg = 0.5
```

```
            kCProd = 0.6
            A = 0
            B = 0
            C = 0

            AProd: => A; cell*vAProd
            ADeg: A =>; cell*kADeg*A
            BProd: => B; cell*kBProd*A
            BDeg: B => ; cell*kBDeg*B
            CProd: => C; cell*kCProd*A*B
            CDeg: C => ; cell*kCDeg*C
        end
        """
    )

print(mod1)
print(mod2)
print(mod3)
```

```
Model(name=negative_feedback, time_unit=s, volume_unit=l, quantity_unit=mol)
Model(name=positive_feedback, time_unit=s, volume_unit=l, quantity_unit=mol)
Model(name=feed_forward, time_unit=s, volume_unit=l, quantity_unit=mol)
```

1.3.1 Simulate the Models

```
In [2]: for mod in [mod1, mod2, mod3]:
            TC = tasks.TimeCourse(mod, end=100, intervals=100, step_size=1)
            viz.PlotTimeCourse(TC, separate=False)
```

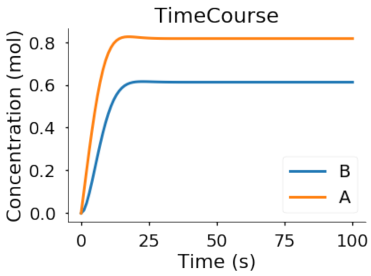

Negative Feedback Model

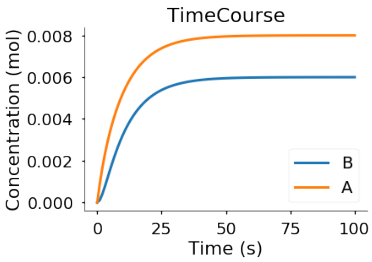

Positive Feedback Model

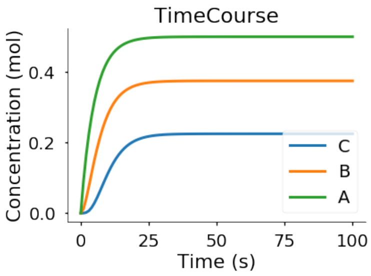

Feed Forward Model

1.3.2 Simulate Synthetic Data

Here we simulate some data from the negative feedback model. This data is automatically saved as a report in the same directory as the model. COPASI time course output automatically contains square brackets around species names (i.e. [A]). This is incompatible with PyCoTools since we need an exact match between model variables and the headers in the experimental data. The `utils.format_timecourse_data` function takes a COPASI formatted data file and formats the data for use in parameter estimation.

```
In [3]:
TC = tasks.TimeCourse(mod1, end=100, intervals=10, step_size=10)
utils.format_timecourse_data(TC.report_name)
```

Out[4]:

	Time	B	A	kBDeg	kBProd	kADeg	vAProd
0	0	0.000000	0.000000	0.4	0.3	0.2	0.1
1	10	0.464639	0.748359	0.4	0.3	0.2	0.1
2	20	0.613698	0.823275	0.4	0.3	0.2	0.1
3	30	0.613132	0.816723	0.4	0.3	0.2	0.1
4	40	0.612348	0.816455	0.4	0.3	0.2	0.1
5	50	0.612369	0.816496	0.4	0.3	0.2	0.1
6	60	0.612373	0.816497	0.4	0.3	0.2	0.1
7	70	0.612372	0.816497	0.4	0.3	0.2	0.1
8	80	0.612372	0.816497	0.4	0.3	0.2	0.1
9	90	0.612372	0.816497	0.4	0.3	0.2	0.1
10	100	0.612372	0.816497	0.4	0.3	0.2	0.1

1.3.3 Perform Model Selection

The model selection problem is defined by providing the path to the folder containing the model and data files to `tasks.MultiModelFit` as a string. A new folder is created for each model which is automatically configured for parameter estimation using settings defined in the arguments to `tasks.MultiModelFit`. Here, we use the genetic algorithm with a population size of 30 over 60 generations. Forty parameter estimations are run for each model (copy_number · pe_number) in serial `run_mode=True` or parallel `run_mode='parallel'`. The `metabolites`, `global_quantities` and `local_parameters` keywords accept lists of strings describing which parameters to be estimated. Here, since these are left at default, all model parameters are estimated between the boundaries of 1^{-2} and 1^2.

```
In [4]: MMF = tasks.MultiModelFit(model_selection_dir,
                                  method='genetic_algorithm',
                                  population_size=30,
                                  number_of_generations=60,
                                  copy_number=2,
                                  pe_number=20,
                                  run_mode=True,
                                  overwrite_config_file=True,
                                  lower_bound=1e-2,
                                  upper_bound=1e2)
        MMF.write_config_file()
```

```
MMF.setup()
MMF.run()
```

1.3.4 Calculate and Plot Model Selection Criteria

To calculate model selection criteria, use the `viz.Model_Selection` class. This saves the model selection data to file and produces violin plots comparing the models. In a violin plot:

- The central spot is the median of the distribution
- The thin line down the middle represents the 95% confidence interval
- The thick central line represents the interquartile range
- The width of the violin is a kernel density estimation representing the shape of the data

Since this function in PyCoTools uses the `seaborn.Violinplot`, users are referred to the seaborn documentation for more details (https://seaborn.pydata.org/generated/seaborn.violinplot.html).

In [5]:
```
## Automatically produces Violin Plots and saves a
## csv file containing model selection criteria
MS = viz.ModelSelection(MMF, savefig=True)
```

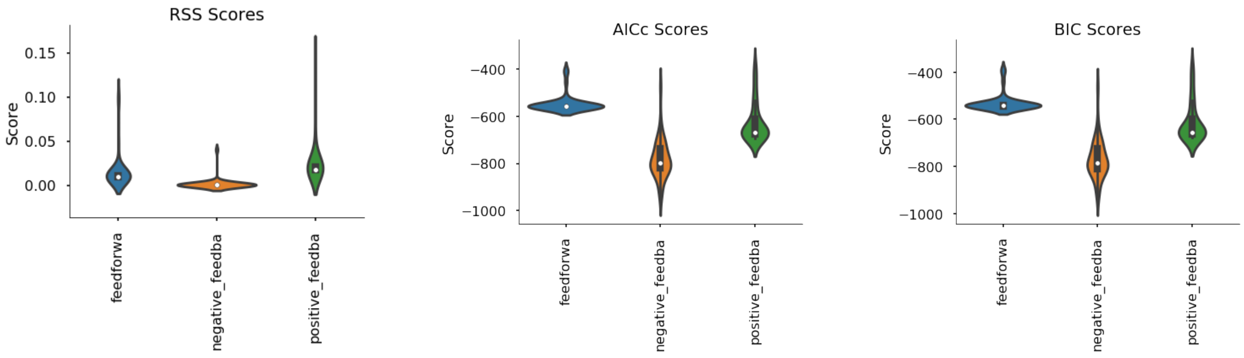

The model selection criteria is available from the `ModelSelection.model_selection_data`.

In [6]:
```
MS.model_selection_data.head()
```

Out[8]:
```
            feedforward                                negative_feedback              \
                    RSS         AICc          BIC             RSS         AICc
RSS Rank
0          0.009863 -560.367290 -543.660398        0.000035 -941.415291
1          0.009918 -560.000346 -543.293453        0.000051 -916.067018
2          0.009934 -559.896087 -543.189194        0.000061 -903.795657
3          0.009938 -559.868720 -543.161827        0.000092 -876.664332
4          0.009942 -559.844882 -543.137990        0.000118 -860.659438

                        positive_feedback
                  BIC              RSS         AICc          BIC
```

```
RSS Rank
0       -929.277363       0.001443 -693.205594 -678.878011
1       -903.929090       0.001572 -687.566491 -673.238908
2       -891.657729       0.001586 -686.989696 -672.662112
3       -864.526404       0.001630 -685.202219 -670.874636
4       -848.521509       0.001735 -681.055345 -666.727762
```

1.4 Interpretation of the Model Selection Problem

While the differences in the best achieved object function value (RSS) are comparable, the negative feedback model reached lower values more often, indicating that more parameter sets represent the data better more often than that of the other models. It should be noted here that the algorithm settings in the configuration were chosen so that the distributions of model selection criteria maintain some variability that are visible in the violin plots. Increasing the stringency of the algorithm by increasing the `population_size` and `number_of_generations` parameters to the genetic algorithm, will increase the separation between the RSS scores in favour of the negative feedback model and cause the violin plots to 'flat line'. This is unsurprising since the data was simulated from the negative feedback model.

The model selection scores are very similar in this instance with both AICc and BIC scores in agreement that simulations produced from the negative feedback model better represent the experimental data.

1.5 Retrieving Best Parameters

Before moving on to visualisation plots and profile likelihoods, we first compare the original parameters from the negative feedback model and with the best estimated values.

The original parameters were:

- A = 0
- B = 0
- kADeg = 0.2
- kBDeg = 0.4
- kBProd = 0.3
- vAProd = 0.1

The `tasks.MultiModelFit` object is an iterable object, much like a python dictionary. The keys of this dictionary are the absolute paths to the COPASI files used in the estimations while the values are the corresponding `tasks.MultiParameterEstimation` objects. To simplify retrieval of the path to the negative feedback model, use the `keys` method of the `tasks.MultiModelFit` object.

```
In [7]: ## get key for negative feedback model
MMF.keys()
```

```
Out[8]: ['/ModelSelectionDemonstration/ModelSelection/negative_feedback/negative_feedback.cps',
ModelSelectionDemonstration/ModelSelection/positive_feedback/positive_feedback.cps',
ModelSelectionDemonstration/ModelSelection/feedforward/feedforward.cps']
```

Here, however since we defined the path to the negative feedback model as `copasi_file1` earlier, we can just use this instead. Once we have a `tasks.MultiParameterEstimation` object, it can be passed to the `viz.Parse` object to retrieve the best parameter estimates as a `pandas.DataFrame`. The `data` attribute of the `viz.Parse` class stores this data.

The best estimated parameters for negative feedback model were:

```
In [8]: ## Parse parameter estimation data into pandas.DataFrame
negetive_feedback_MPE = MMF[copasi_file1]
data = viz.Parse(negetive_feedback_MPE).data
data.iloc[0]
```

```
Out[8]:
A          0.012177
B          0.011752
kADeg      0.202126
kBDeg      0.398976
kBProd     0.301419
vAProd     0.100330
RSS        0.000030
Name: 0, dtype: float64
```

The kinetic parameters were estimated accurately but the initial concentration parameters were more difficult. This is because the initial values were 0 in the model which is more difficult to estimate. Generally, an initial concentration parameter of 0 will always be difficult to estimate and it should be avoided.

1.6 Visualising Parameter Estimation Data From a Model Selection Problem

Often it is useful to dig deeper into a model selection problem to analyse individual models separately. This can be done by iterating over the 'tasks.MultiModelFit' object to get a handle on the underlying `tasks.MultiParameterEstimation` objects that were used for the estimations. Classes in the `viz` module are designed to operate on the `tasks.MultiParameterEstimation` object, thereby enabling us to efficiently produce diagnostic plots for each sub-parameter estimation problem.

1.6.1 Ensemble Time Courses

```
In [9]: for MPE in MMF:
            viz.PlotTimeCourseEnsemble(MPE)
```

Negative Feedback Simulations

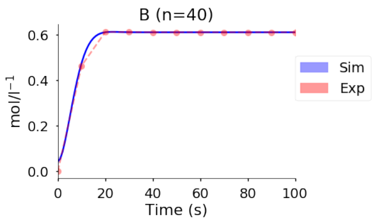

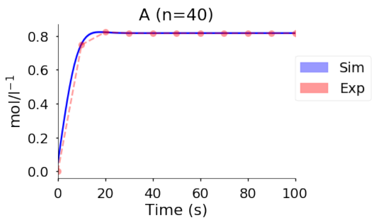

Positive Feedback Simulations

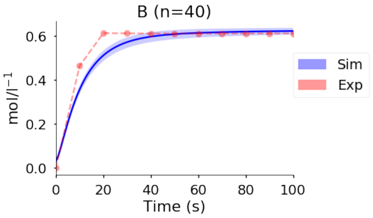

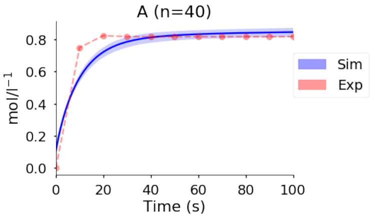

Feedforward Simulations

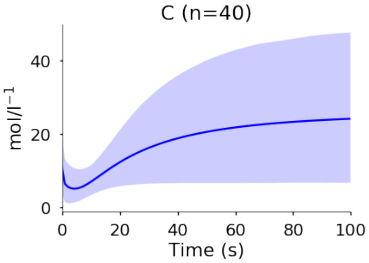

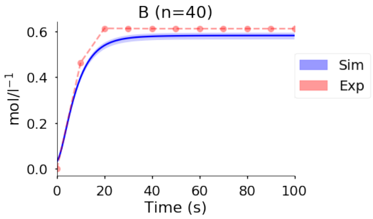

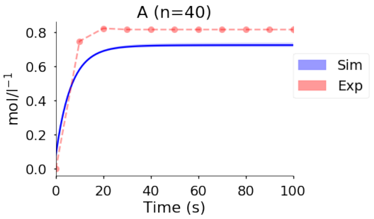

1.6.2 Likelihood ranks

```
In [10]: for MPE in MMF:
             viz.LikelihoodRanks(MPE)
```

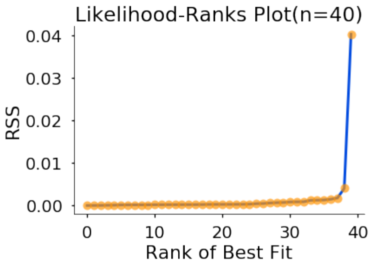

Negative Feedback

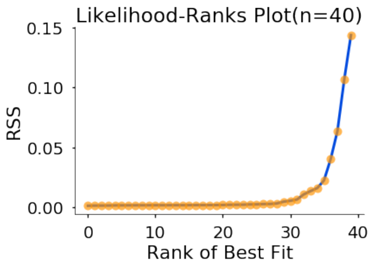

Positive Feedback

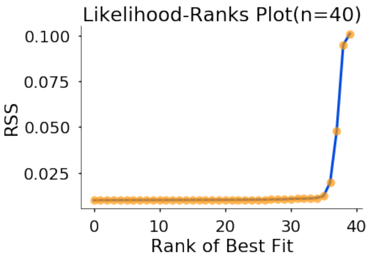

Feedforward

1.6.3 Boxplots

```
In [11]: for MPE in MMF:
             viz.Boxplots(MPE, num_per_plot=10, log10=True)
```

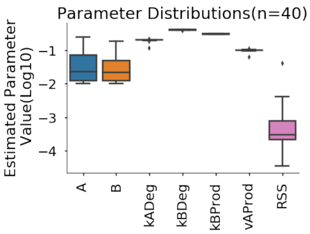

Negative Feedback

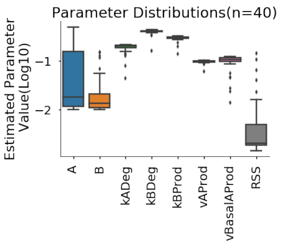

Positive Feedback

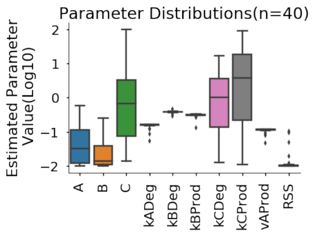

Feed Forward

1.6.4 Correlations

In the remainder of this document, we isolate the negative feedback model to reduce the number of graphs produced. Note, however, that these graphs can be produced for each model by iterating over the MMF variable as above.

```
In [12]:
## get underlying MultiParameterEstimation object
MPE = MMF[copasi_file1]
```

Pearson's Correlations Looking at all combinations of two parameters using scatter graphs can be overwhelming. In some instances, two parameters are linearly related and can be efficiently identified using the Pearson's correlation.

```
In [13]:
## look at pearsons correlation between parameters
viz.PearsonsCorrelation(MPE)
```

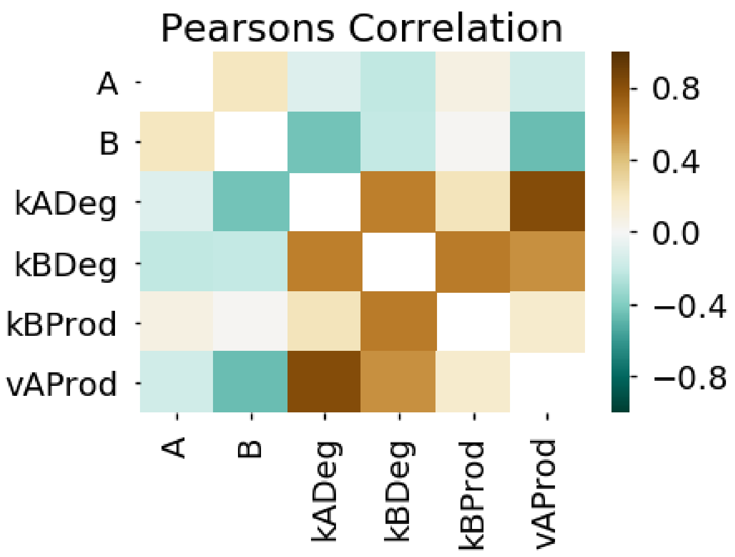

While useful in searching for the most obvious (linear) relationships, Pearson's correlation should be used with caution in the non-linear ODE model setting because which the relationships between parameters are generally more complex. Also, parameter values may be pinpointed within a very specific region of parameter space and yet still appear highly correlated.

Scatter Graphs All combinations of scatter graph can be produced by replacing the x and y arguments to the viz.Scatters class with 'all'. Here however we restrict the number of plots to four.

```
In [14]: viz.Scatters(MPE, x=['kADeg', 'kBDeg'], y=['kBProd', 'kBDeg'])
```

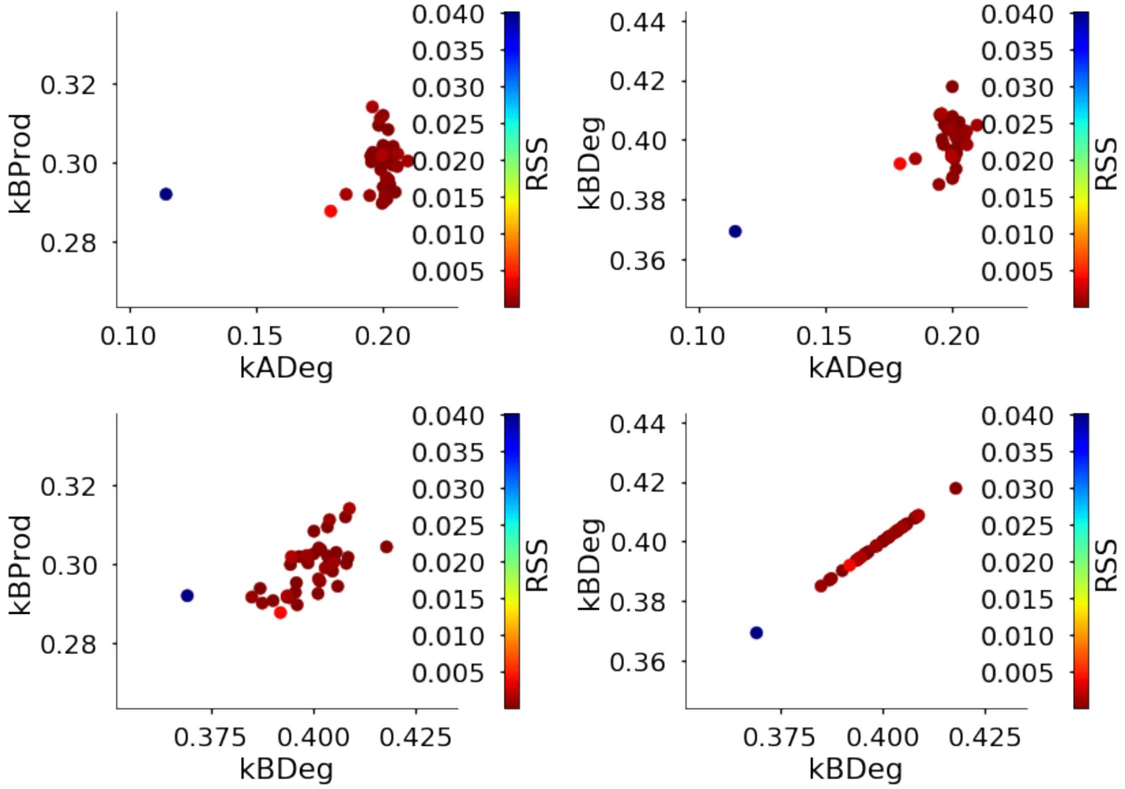
kBProd
kADeg
RSS
kBDeg
0.040
0.035
0.030
0.025
0.020
0.015
0.010
0.005
0.32
0.30
0.28
0.44
0.42
0.40
0.38
0.36
0.10
0.15
0.20
0.375
0.400
0.425

1.6.5 Profile Likelihoods

Detailed information about profile likelihoods can be found in the main text and in Raue *et al.* (2009). To calculate them using PyCoTools and COPASI, first ensure you have performed parameter estimation and your model is a a minimum with respect to the data. `tasks.ProfileLikelihood` expects a model to already be configured for parameter estimation (which is usually the case). The `tasks.ProfileLikelihood` class creates a folder for each parameter set specified with the `index` keyword (which can be a list of integers) and a COPASI file for each parameter within that parameter set. Each model is named after the parameter of interest and is used to calculate a profile likelihood for that parameter. In each profile likelihood, the independent variable or parameter of interest is systematically varied in a parameter scan of parameter estimations. The variable of interest is fixed during the parameter estimation all other parameters are allowed to vary (e.g. re-optimised). The path traced by the objective function value over the course of this scan is the profile likelihood.

Calculation

```
In [15]: pl = tasks.ProfileLikelihood(
                        MPE.model,
                        parameter_path=MPE.results_directory,
                        index=[0, 1],
                        run=True,
                        intervals=20
                )
```

Visualization

```
In [16]: viz.PlotProfileLikelihood(pl, title='', interpolation='cubic')
```

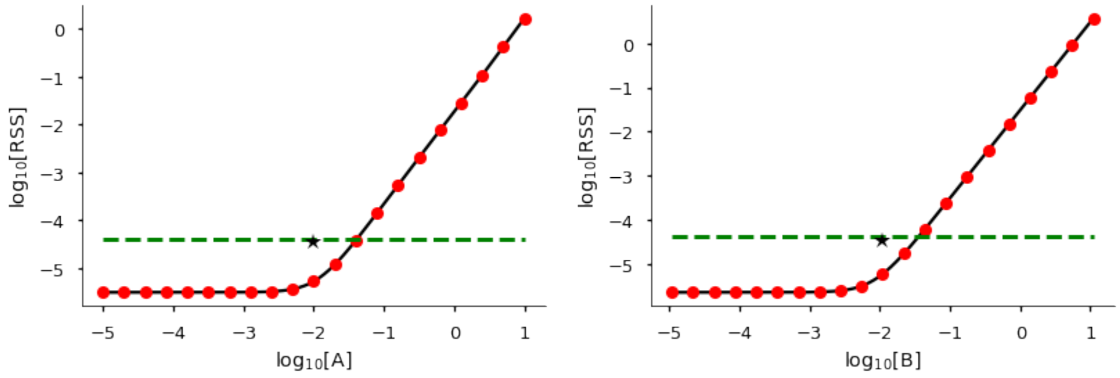

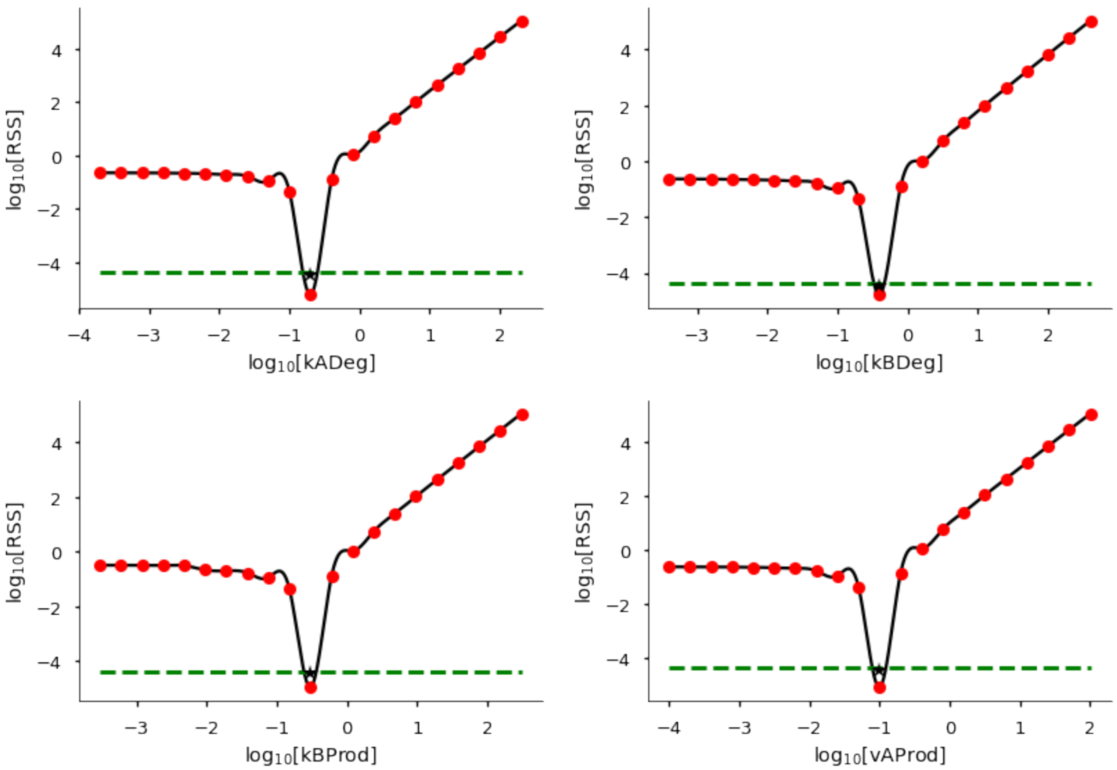

Interpretation All kinetic parameters are identifiable to a high degree of confidence. The initial concentration parameters however are practically non-identifiable.

1.6.6 Model Reduction

To plot the traces of other parameters along the course of the profile likelihood, provide the name of the parameter you would like to include as arguments to the `x` and `y` arguments. In general these graphs are difficult to interpret but may be of use for directing strategies of model reduction. For more details, please see Maiwald *et al.* (2016).

```
In [17]: viz.PlotProfileLikelihood(pl,
                x=['kADeg','kBDeg', 'A', 'B'],
                y=['kBProd', 'A', 'B'],
                interpolation='cubic',
                title='')
```

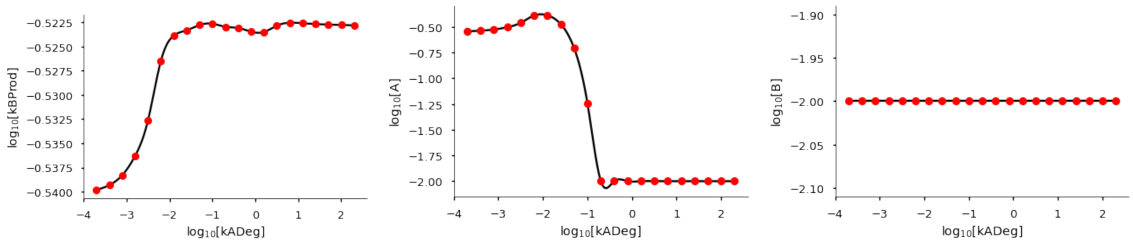

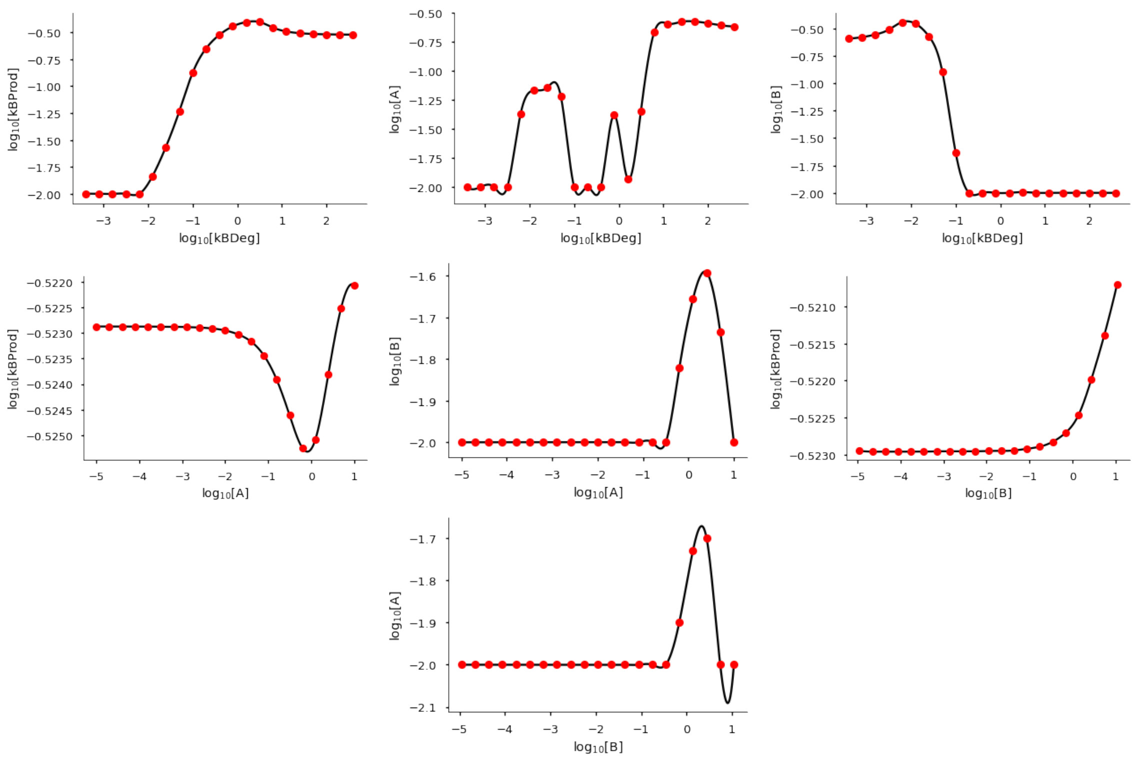

References

Maiwald, T., Hass, H., Steiert, B., Vanlier, J., Engesser, R., Raue, A., Kipkeew, F., Bock, H. H., Kaschek, D., Kreutz, C., *et al.* (2016). Driving the model to its limit: Profile likelihood based model reduction. *PloS one*, **11**(9), e0162366.

Raue, A., Kreutz, C., Maiwald, T., Bachmann, J., Schilling, M., Klingmüller, U., and Timmer, J. (2009). Structural and practical identifiability analysis of partially observed dynamical models by exploiting the profile likelihood. *Bioinformatics*, **25**(15), 1923–1929.

Welsh et. al., PyCoTools: A Python Toolbox for COPASI. Supplementary Figures.

April 20, 2018

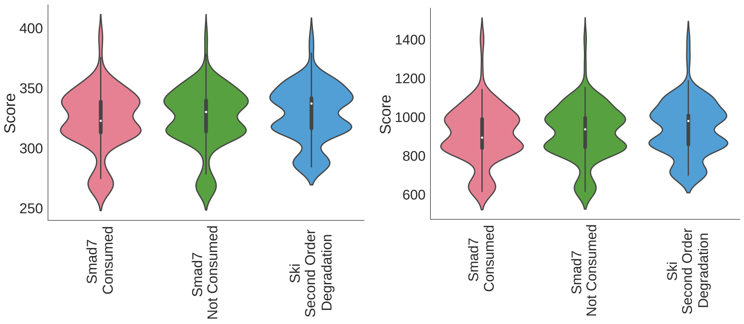

(a) BIC

(b) RSS

Figure S1: Violin plots depicting distributions of Bayesian information criteria (BIC) and residual sum of squares (RSS) values for each model

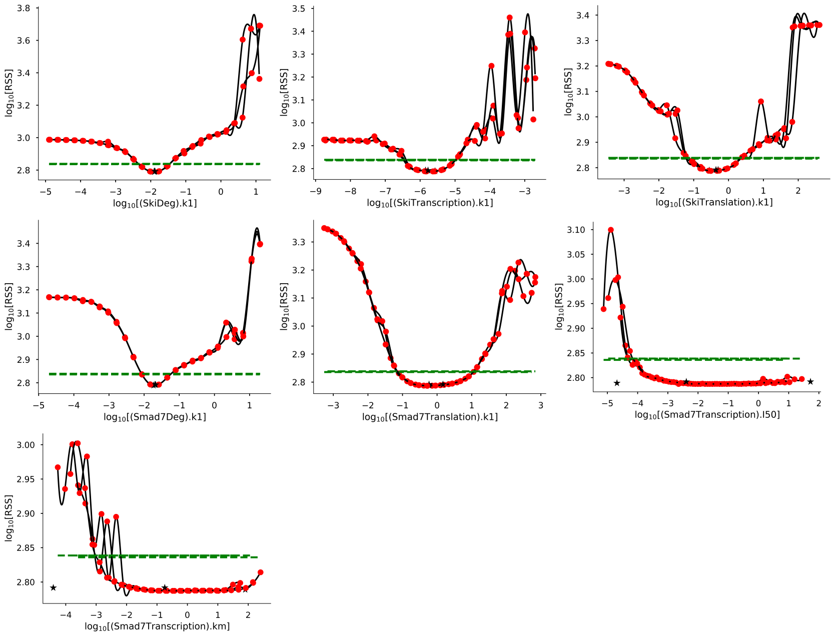

Figure S2: Profile likelihoods for 7 of 10 estimated parameters for Model 2. All parameters shown are identifiable except the I50 and km parameters of the 'Smad7Transcription' reaction, which are practically non-identifiable. Profile likelihoods were calculated from the top three best parameter sets. Black stars indicate the best parameter values from the original parameter sets while the red spots mark the best objective function value after re-optimisation. The lines between red spots have been interpolated with a cubic spline. The dotted green line indicates the 95% confidence level.

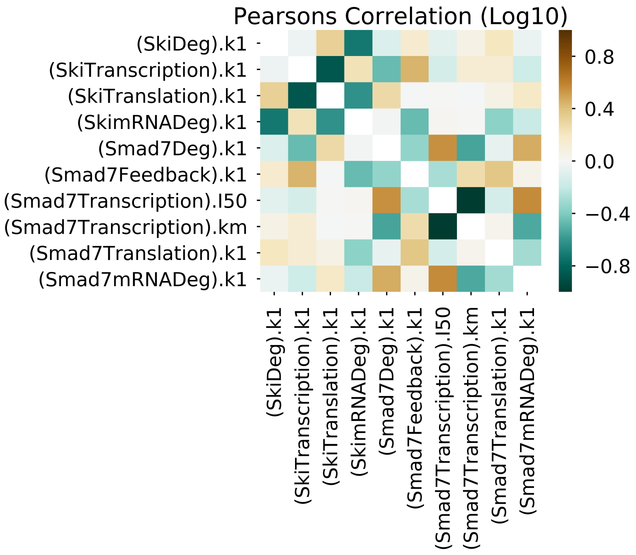

Figure S3: Pearson's correlation computed on a log10 scale between all 2-way combinations of parameters in the top 40 parameter sets in model 2. This graph was produced using the 'viz.PearsonsCorrelation' class.

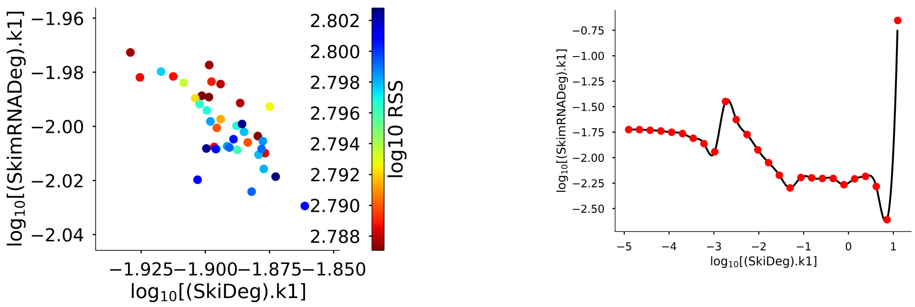

(a) Scatter graph.

(b) Profile likelihood

Figure S4: (a) Scatter graph and (b) profile likelihood trace of the '(SkiDeg).k1' parameter on the x-axis and '(SkimRNADeg).k1' parameter on the y-axis.

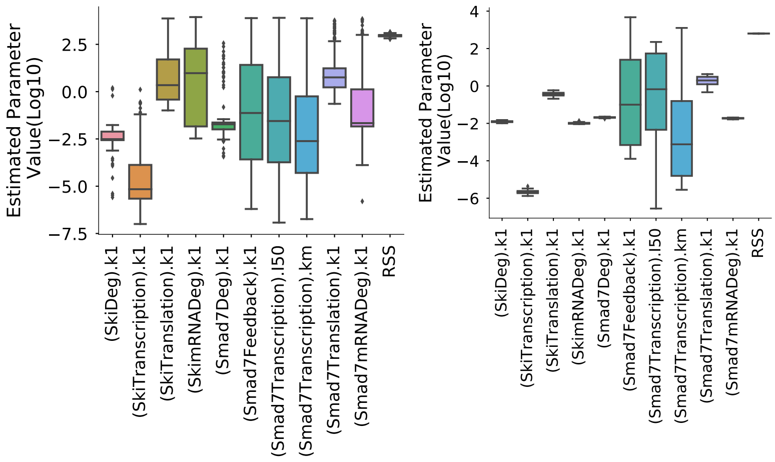

(a) Smad7 consumed (n=300) (b) Smad7 not consumed (n=30)

Figure S5: Box plots showing parameter distributions for each parameter in the 'Smad7 consumed topology' (Model 2). A comparison is made between (a) the distributions of each parameter for all 300 and (b) the distributions of the top 10 percent of parameter sets only.

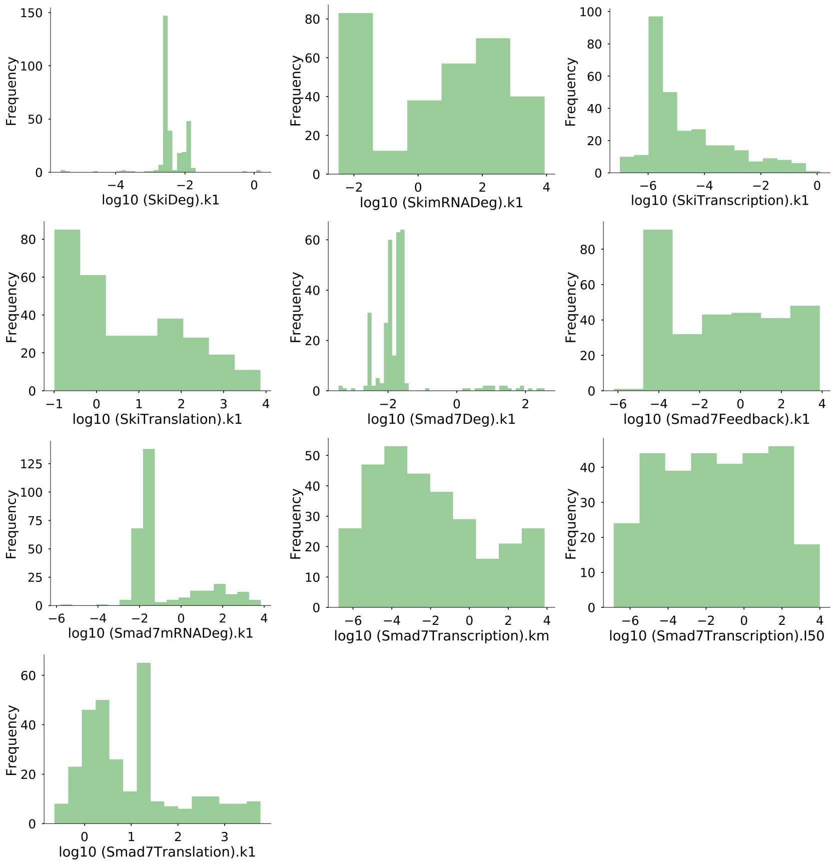

Figure S6: Histograms showing parameter distributions for each parameter in the 'Smad7 consumed topology' (Model 2)
